# Reproductive Biology of *Rheum webbianum* Royle, a Vulnerable Medicinal Herb From Alpines of North-Western Himalaya

**DOI:** 10.3389/fpls.2022.699645

**Published:** 2022-02-17

**Authors:** Ishfaq Ahmad Wani, Susheel Verma, Parvaiz Ahmad, Hamed A. El-Serehy, Maha J. Hashim

**Affiliations:** ^1^Conservation and Molecular Biology Lab, Department of Botany, Baba Ghulam Shah Badshah University, Rajouri, India; ^2^Department of Botany and Microbiology, King Saud University, Riyadh, Saudi Arabia; ^3^Department of Zoology, College of Science, King Saud University, Riyadh, Saudi Arabia; ^4^Department of Bioscience, University of Nottinghamshire, Nottingham, United Kingdom

**Keywords:** *Rheum webbianum*, hermaphrodite, protandry, incurved stigma, mixed mating, ambophilous, outcrossing

## Abstract

Information on reproductive biology and pollination ecology studies of threatened plants are essential to develop strategies for their sustainable utilization and effective conservation. As such, these studies were conducted on *Rheum webbianum*, a high-value “vulnerable” medicinal herb of the north-western Himalaya. This species presents a unique mode of reproductive behavior through the involvement of different floral events, including the movement of reproductive organs. The plants survive extremely cold conditions through underground perennating rhizomes that sprout into juvenile shoots with the onset of the favorable climatic conditions. The peduncle arises from the axils of the radical leaves, bearing a globular collection of densely arranged hermaphrodite flowers with temporally separated male and female phases; the male phase precedes the female phase (protandry). Anther dehiscence and stigma receptivity is post-anthesis. Anthers dehisce longitudinally along margins, liberating a large mass of spherical and tricolpate pollen with spinulose exine. Pollen viability decreased to < 10% on day 9. Pistil is tristylous, with each style terminating into a fan-shaped stigma lobe. The pollen receptive surface of each stigmatic lobe remains incurved at an angle of 360° and shows upward movement after anthesis, forming a funnel-like structure at an angle of 180° with respect to the ovary. Pollination syndrome is ambophilous. Spontaneous autogamy or geitonogamy to a certain extent is achieved in this species due to the arrangement of flowers in the inflorescence and overlapping of male and female reproductive phases among them. Incurved stigmatic lobes and outward movement of stamens too facilitate outcrossing. Pollen/ovule ratio estimates, results of pollination experiments on breeding behavior, outcrossing, and self-compatibility indices demonstrated that plants are self-compatible and cross-fertile.

## Introduction

The genus “*Rheum*” belongs to the family Polygonaceae and was first reported by Carl Linnaeus in 1753 (Linnaeus, 1753). Globally, this genus is represented by about sixty perennial species distributed in the mountains of Asia and Europe (Kao and Cheng, 1975; Li, 1998). In the Indian subcontinent, this genus is represented by seven species (Ganie et al., 2014), all of them are present in Kashmir Himalaya (Stewart, 1972). *Rheum webbianum* is an important species of this genus inhabiting alpine regions between 2,400 and 4,300 m.a.s.l (Agarwal et al., 2001; Rashid et al., 2014; Tabin et al., 2016). Commonly known as “Pambhak” (leaves) or “Pambchalan” (rhizome), it is medicinally important finding wider utilization in the pharmaceutical sector in the preparation of drugs combating cancer (Srinivas et al., 2007) and body cholesterol (Abe et al., 2000). It is also highly important in treating indigestion, abdominal disorders, boils, wounds, and gastritis through traditional medicinal practices (Chaurasia and Ballah, 2009; Tabin et al., 2016). Incessant exploitation of the plant along with cattle grazing and trampling has threatened its existence in nature. Unprecedented overexploitation has squeezed its populations and as such, it has been listed as a vulnerable medicinal herb from North–West Himalaya (CAMP, 2003; Ved et al., 2003; Tali et al., 2014). In Kashmir Himalaya, which constitutes a part of North-West Himalaya, *R. webbianum* grows at higher elevations between 2,836 and 4,497 m.a.s.l. (Wani et al., 2021).

It has been observed more frequently that at higher elevations, plants exhibit greater phenotypic plasticity as it enables them to adapt to the highly variable environment there (Stocklin et al., 2009; Pluess et al., 2011). Plants adjust key physiological and reproductive processes (Korner et al., 1989; Becker et al., 2006; Korner, 2007) as well as morphological traits (Phillips et al., 2011) in response to the climatic shifts. It has also been reported that phenotypic plasticity allows to compensate the short growing season of plants by allowing rapid reproduction in some alpine and subalpine species (Stinson, 2004). Reproduction is an important and fragile step in the life history of the plants and a prerequisite natural process to multiply, evolve, and survive (Li et al., 2018). Therefore, detailed knowledge on different attributes of reproductive biology (flowering phenology, breeding system, pollinator availability, pollination and reproductive success, seed and fruit formation, seed germination and seedling recruitment) is indispensable for determining the nature of species adaptation. Such studies bring into account the reproductive bottlenecks of plants and greatly aid in designing effective conservation strategies (Moza and Bhatnagar, 2007; Bentos et al., 2008; Kaur et al., 2013; Gopalakrishnan and Thomas, 2014; Sreekala, 2017). These studies also provide valuable information on speciation, adaptation, hybridization, and systematics (Anderson et al., 2002; Neal and Anderson, 2005). Mating and breeding systems are the integral components of plant reproduction as they determine the genetic structure of populations (Schemske and Lande, 1985) and also drive the evolutionary changes in closely related species (Brauner and Gottlieb, 1987; Ashman, 2003).

There is ample information that plant-pollinator interaction determines the success of sexual reproduction (Gan et al., 2013; Castano et al., 2014) and understanding of seed biology (germination and development) is important in the quality improvement and cultivation of medicinal plants (Bareke, 2018). While a large group of pollinators is generalists (Ollerton, 2014), there are some specialists, which have evolved throughout the course of evolutionary history (Proctor et al., 1996; Endress and Bruyns, 2000). Loss of variability among the populations and inbreeding depression are the inevitable consequences of self-pollination. Outcrossing is believed to be the most evolved form of breeding system (Evans et al., 2003; Mayer et al., 2011; Nebot et al., 2016; Sreekala, 2017). Several plant species are known to possess specific adaptive strategies to combat the loss of pollinators and to modify their breeding system toward a more favorable mode, that is, outcrossing. In certain cases, selfing is avoided by plants to promote outcrossing through stylar movement (Verma et al., 2004). Under certain limiting circumstances when pollination fails, the plants move their styles to get pollinated and assure reproduction (Ruan et al., 2009; Khajuria et al., 2011).

The present study was therefore carried out to understand the variation in growth characteristics, reproductive success, and pollinator behavior of *R. webbianum* in relation to the habitat variability at high altitude areas of Zanskar and Pir Panjal mountain ranges of Kashmir Himalaya. This study also aimed at designing strategies for conservation, cultivation, and sustainable use of wild populations, as well as to identify the most suitable and productive habitats for the growth of *R. webbianum*.

## Materials and Methods

### Study Area and Plant Identification

The present study was conducted from May 2017 to September 2020 at the Zanskar and Pir Panjal subranges of the north-western Himalaya extending through Ladakh and Kashmir union territories (UTs) of India. Fieldwork was carried out in three different regions: Panikhar, Kargil (PK) (34°07′08.442°N; 75°57′06.254°E), Katarkhal, Shopian (KS) (34°13′.721°N; 75°57′.912°E), and Tiken Batpora, Pulwama (TBP) (33.8481° N, 74.8697°E). The population at Panikhar lies in the Zanskar region and was located at an altitude of 3,327 m.a.s.l. Katarkhal lies in the Pir Panjal mountain range and the population was located at an altitude of 3,474 m.a.s.l. The population at Tiken Batpora was an introduced population, which was maintained in a conservatory under *ex situ* conditions. At all the sites, the species was studied throughout its different phenophases, and necessary data were recorded and subjected to further analysis.

The species was identified by consulting the herbarium specimens and published data. The specimens collected during the present study were compared with the specimens submitted under voucher numbers 19293, 31398, 31399 at Herbarium of Department of Botany, Punjabi University, Patiala and 31204 at Kashmir University Herbarium. Earlier published records related to morphology and distribution of this species were also referred for identification (Sagoo and Farooq, 2011; Baig et al., 2014; Tali et al., 2014; Tabin et al., 2016; Ganie et al., 2017).

### Phenology

The data were collected on different phenophases immediately after the sprouting of rhizomes until senescence in all 3 populations. Phenological events (sprouting, vegetative phase, flowering, fruiting, seed maturation, and senescence) were monitored on tagged plants (seven plants from each site) at their three locations on a population basis. During the flowering period, observations were carried out on a daily basis, while for fruit and seed traits, the observations were carried out once a week (Tandon et al., 2003; Verma et al., 2003, 2004; Smitha and Thondaiman, 2016).

### Floral Traits and Biology

Twenty-one mature and healthy plants (7 from each population) with full bloom were randomly selected and tagged to study floral traits and biology. A detailed study regarding different quantitative characteristics of floral parts, namely, length and width of the anther, stamen length, stigma length and width, ovary length, ovary width, and the size of ovule, was carried out under a stereo zoom microscope (Olympus- BX51). The number of flowers per inflorescence, number of anthers per flower, length of inflorescence, and length of the peduncle were noted directly in the field with applied SDs.

Principal component analysis (PCA) was performed to investigate whether the plant populations sampled at the three sites differed in the studied morphological and reproductive characters. It simplifies the complexity of data set by reducing it to those variables that show a higher contribution to give the best summary of the data using a limited number of PCs. We used the “*stats”* package for PCA analysis and “*ggbiplot”* for plotting the respective ordination biplot. All the analyses were performed in R software v.4.0.3 (R Core Team, 2020), using the packages cited within.

### Anthesis Time and Direction of Anther Dehiscence

The time of blooming of individual flowers and inflorescences was observed on open flowers from all the populations. Filament movement and anther dehiscence were observed on the same flowers (*n* = 20) with the help of a hand lens and protector (Nautiyal et al., 2009; Li et al., 2018). The direction of anther dehiscence with respect to the stigma was determined as the degree of movement of the filaments of the bithecous anthers relative to their initial position.

### Pollen Stainability, Viability, and the Pollen-Ovule Ratio

The stability and viability of pollen were determined using 1% Fluorescein diacetate (FDA) and acetocarmine and (Shivanna and Rangaswamy, 1992). The number of viable and non-viable pollen grains was counted under a Nikon 80i eclipse microscope at 10x magnification. The pollen-ovule ratio was calculated by counting the number of pollen grains per anther and then multiplying the figure by the number of anthers per flower. The ovule count was determined by treating ovaries with 4N NaOH for 12–14 h at 60°C and washing them to remove traces of NaOH. Finally, the ovaries were squashed in Lewis stain and visualized under a microscope. The pollen-ovule ratio was calculated following Verma et al. (2008).

### Stigma Receptivity

The stigma receptivity was checked by fixing hand-pollinated stigmas of different ages (21 stigmas from 7 plants) in Carnoy’s fixative containing absolute alcohol and glacial acetic acid at a ratio of 3:1. After a predefined time (2–4 h), these stigmas were transferred to 70% alcohol until further use. For microscopic examination, pistils were stained with Lewis stain (mixture of 2 ml of 1% aq. Acid fuchsin, 2 ml of 1% aq. light green and 40 ml lactic acid and 46 ml distilled water) (Lewis, 1979) and in aniline blue for fluorescence microscopy (Shivanna and Rangaswamy, 1992; Khajuria, 2013). The stigmas that showed pollen germination were considered receptive.

The hydrogen peroxide (H_2_O_2_) method was also used to determine stigma receptivity, wherein bubbling in the presence of H_2_O_2_ is considered to be a positive result (Li et al., 2018). Twenty-one stigmas from 7 plants were subjected to the treatment to determine the stigma receptivity.

### Breeding Behavior

To understand the breeding system in *R. webbianum*, healthy buds that would definitely bloom were selected randomly and tagged. At flower anthesis, six different manual pollination treatments were executed.

#### Apomixis or Agamospermy

During bud condition or at the time of anthesis, emasculation was performed in buds (*n* = 150) for each population followed by bagging with butter paper bags (1.5 × 2 cm) to prevent pollen deposition by wind or insects. The tagged buds were regularly monitored for fruit set, if any.

#### Unassisted Pollination, Spontaneous Autogamy, or Autonomous Selfing

Flower buds were bagged (*n* = 150) for each population for unassisted pollination. The bags were removed after the completion of the reproductive phase.

#### Selfing or Facilitated Autogamy

Emasculated flowers were manually pollinated with pollen of the same flower followed by bagging.

#### Geitonogamy

Emasculated flowers were manually pollinated with the pollen of other flowers from the same plant, followed by bagging.

#### Xenogamy

Emasculated flowers were bagged until the stigmas of these flowers were manually pollinated with pollen collected from different individuals of the same population. Such flowers were bagged to avoid any contamination from self or geitonogamous pollen.

#### Control

The flowers were kept as such for open pollination and observed for fruit and seed set after flowering was complete.

Two-way ANOVA was performed to evaluate simultaneously the effect of site and treatment on the development of fruit and seed.

### Outcrossing Index, Index of Self-Incompatibility, and Inbreeding Depression

The outer crossing index (OCI) was calculated following the methods of Dafni (1992) and Barman et al. (2018) (Table 1). The mean scores of flower diameter, placement of anthers and stigma, and time of anther dehiscence and stigma receptivity were added to obtain the possible results (Table 2).

**TABLE 1:** Observations of different floral parameters to determine outcrossing index of *Rheum webbianum* (following Dafni, 1992).

Observation	Expression	Value
Flower diameter	< 1 mm	0
	1–2 mm	1
	2–6 mm	2
	> 6 mm	3
Anther dehiscence and stigma receptivity	Homogamy	0
	Protogyny	0
	Protandry	1
Position of stigma and anthers	Anthers and stigma at the same level	0
	Separated and contact not possible	1

**TABLE 2:** Outcrossing index value *vis-a-vis* type of breeding system (following Dafni, 1992).

Outcrossing index value	Breeding system (flower diameter + anther dehiscence and stigma receptivity + Position of stigma and anthers)
0	Cleistogamy
1	Obligate autogamy
2	Facultative autogamy
3	Predominantly self-pollinated
4	Mixed mating
5	Predominantly cross-pollinated

The index of self-incompatibility was determined by calculating the percentage of average fruit set from manual self-pollination to manual cross-pollination. Values below or equal to 0.2 depict self-incompatibility and values greater than this level show self-compatibility. Inbreeding depression was calculated following the method of Ramirez and Nassar (2017). The difference in the level of inbreeding depression between the three sites was ascertained through one-way ANOVA and will help us to analyze the loss of vigor with respect to different pollination treatments.


ID=1-(ωs/ωo)

Where ωs = mean seed weight of self-pollinated seeds; ωo = mean seed weight of cross-pollinated seeds.

### Seed Germination

The dry weights of 6-month-old seeds (50 seeds with four replicates from each site) obtained from auto-, geitono-, and xenogamy were recorded. The seeds were soaked in water at 19 ± 1.7°C, removed at equal intervals, wiped dry, weighed, and soaked again until a constant weight was obtained. The seeds were subsequently placed in plastic plates [22.5 × 4 cm (l × h)] containing vermicompost and monitored for germinability. Percentage of seed germination and seedling survival were recorded.

### Pollination Ecology

The pollination censuses were conducted at peak flowering periods during three consecutive seasons from 2017 to 2019. The pollination efficiency was measured in terms of pollen load deposited on the stigma of the flowers at the first visit of the pollinator. It was calculated from 30 different flowers (10 each from different populations) following Singh et al. (2014). Pollinated stigmas were stained with 0.2% acetocarmine, and the total number of pollen deposited was checked under a light microscope at 40×. To ascertain anemophily, glass slides smeared with Mayer’s albumen were hung from wooden stands kept around the plants for 24 h and finally observed under a light microscope for pollen deposition (Khajuria, 2013). Entomophily was determined on keen observations in the field for 1 week during peak flowering. A large number of flowers were emasculated at the onset of the night at Panikhar and Tiken Batpora populations and kept unbagged throughout the night. Stigmas of such flowers were clipped early in the morning, fixed in Carnoy’s fixative (3 parts absolute alcohol:1 acetic acid), transferred to 70% alcohol after 4 h, and observed later on for pollen load and germination.

Different pollination indices were calculated for all the pollinators to determine their contribution to pollen transfer. The foraging behavior (FB) of insect visitors was ascertained through direct field visitations at regular intervals of time. It was determined as the time spent by a particular pollinator per inflorescence per visit (Sajjad et al., 2009; Yaqoob and Nawchoo, 2016). Foraging speed (FS) was calculated as the average number of flowers visited per minute of time (Pando et al., 2011; Yaqoob and Nawchoo, 2016). Insect visitation efficiency (IVE) and insect visitation frequency (IVF) were determined following the methodology put forward by Yaqoob and Nawchoo (2016).

The one-way ANOVA test was performed to study the statistical significance of different pollinators in the transfer of pollen and to determine the pattern of pollen deposition on the different body parts of the pollinators.

## Results

### Habitat and Phenology

**FIGURE 1:**
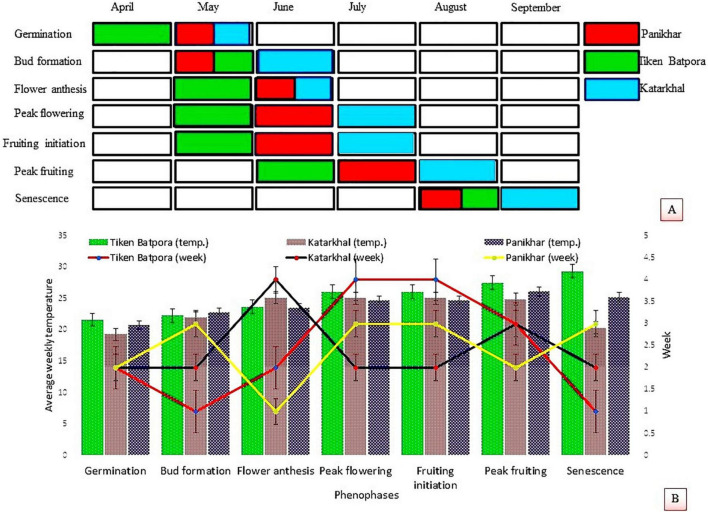
**(A)** Phenophases of *Rheum webbianum* at *in situ* and *ex situ* populations. **(B)** Response of different phenophases to average weekly temperature and time periods.

*Rheum webbianum* Royle (Polygonaceae) is a perennial stout rhizomatous herb that grows on the exposed rocky terrain of alpine and subalpine zones of the Panikhar and Katarkhal regions. Observations pertaining to the time period of leaf flushing, flowering, fruiting, seed formation, and senescence reveal that a plant completes its life cycle within a period of 6–7 months. It overcomes harsh winters in the form of dormant underground rhizomes. With the onset of the spring season, growth resumes and the dormant rootstock sprouts into juvenile shoots. Increased temperature and photoperiod release the dormancy of perennating rhizomes, and the time period of this activity has been observed to be different at different locations. An earlier outburst of cauline leaves was seen in populations growing at TBP compared with populations growing at KS and PK. The plants bear terminal, branched inflorescences that remain enclosed in a sheath before the onset of anthesis, and they bear densely arranged hermaphrodite flowers. The peduncle attains a length of 0.5–1.5 m ± 0.6 feet. The wilting of tepals from their margins and their inward bending movement acts as a signal of phenophase shift from flowering to fructification. As the fruit ripens, the bracts wither leaving behind ragged stems covered with panicles of deep brown pendulous fruits bearing albuminous seeds. Most of the leaves dry after yellowing and remain persistent on the parent plant by thick and leathery petioles. Detailed phenological events are shown in Figures 1A,B.

### Morphometric Analysis, Floral Traits, and Floral Biology

The plants of *R. webbianum* in different populations showed significant differences with respect to their morphological characters. The lowest values for morphological characters were recorded for the KS population growing at the highest elevation and the highest values were observed for the TBP population. The plant body is differentiated into a thick rhizome that penetrates deep into the soil. The size of rhizome varies from 14 to 55 cm. The leaves are dark green, thick, and glabrous and attain the size of 19 ± 8 (width) and 33 ± 13.3 (length) cm. Each flower of *R. webbianum* has creamish white campanulate and hairless perianth represented by six tepals. The lifespan of individual flowers (from anthesis up to swelling of the ovary) and inflorescences was approximately 9 ± 3.02 and 18 ± 3.03 days, averaging across all the sites. The flowers are completely zygomorphic, bisexual, and protandrous. Androecium is represented by 9 stamens with elliptical, bithecous, free or subconnate, pale yellow anthers with filaments inserted on the torus at the base of the perianth. The gynoecium is represented by a single carpel with one ovule. The pistil is tristylous with a single triangular ovary and three erect or deflexed styles, each terminating to a fan-shaped stigma lobe. The nectaries form a ring at the base of the ovary. The fruits are three-sided achenes, oblong or orbicular, notched at both ends. The fruits are light green in color, which turn reddish-brown upon maturity. Complete morphological details of the floral parts are given in Table 3.

**TABLE 3:** Morphometric analysis of floral characters of *Rheum webbianum* across three different study areas.

Floral trait	TBP	PK	KS
Length of a flower	1.5 ± 0.3 cm	1.7 ± 0.3 cm	1.6 ± 0.2 cm
Width of a flower	0.9 ± 0.3 cm	1.1 ± 0.3 cm	1.1 ± 0.2 cm
Tepal length	6 ± 1.4 mm	6 ± 1.6 mm	5 ± 0.3 mm
Tepal width	4 ± 0.9 mm	3.8 ± 0.8 mm	3.9 ± 0.7 mm
Length of anther	594.1 ± 24.60 μm	771.8 ± 17.07 μm	697.9 ± 21.01 μm
Width of anther	307.52 ± 11.09 μm	359.9 ± 15.5 μm	352 ± 7.7 μm
Length of dehiscence line	569.39 ± 4.98 μm	678.45 ± 4.39 μm	672 ± 4.12 μm
Connective length	203 ± 13.93 μm	227.50 ± 21.17 μm	235.14 ± 19 μm
Pollen length	2.571 ± 0.27 μm	3.39 ± 0.22 μm	3.27 ± 0.32 μm
Pollen width	2.12 ± 0.19 μm	2.97 ± 0.25 μm	2.52 ± 0.15 μm
Length of stamen	1.4 ± 0.3 cm	1.7 ± 0.28 cm	2.2 ± 0.35 cm
Stigma lobe (w × b)	469.39 × 518.72 ± 27.7 × 19.9 μm	639.22 × 675.9 ± 56.59 × 47.07 μm	616.89 × 637.7 ± 57.21 × 42.02 μm
Style (l × b)	172.96 × 79.06 ± 21 × 14.05 μm	178.88 × 81 ± 19.5 × 14.09 μm	175.9 × 80.12 ± 21.5 × 19.50 μm
Ovary length (20×)	1329.76 μm	1165.017 μm	1409.58 μm
Ovary width (20×)	945.551 μm	770.796 μm	978.431 μm
Length of ovule	601.552 μm	559. 775 μm	641.730 μm
Width of ovule	361.14 μm	311.283 μm	374.40 μm
Seed length	2 mm	2 mm	2 mm
Seed width	1 mm	1 mm	1 mm
Length of inflorescence	38.2 ± 8.4 inch	32.5 ± 6.6 inch	20.9 ± 4.7 inch
Width of inflorescence	16.7 ± 5.4 inch	14.4 ± 4.7 inch	12.4 ± 4.7 inch
Number of flowers	1,346 ± 303.9	1,098 ± 241.4	784 ± 127.3

**FIGURE 2:**
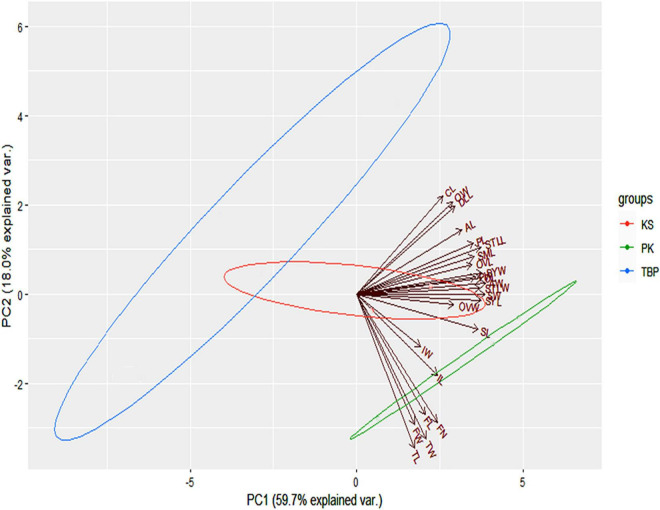
Ordination biplot of the morphological variables at three studied sites. Directions along with the magnitudes of the variables driving each axis are also shown.

Based on the Kaiser–Guttman criterion, only the first two PCs retained the variation greater than the average eigen value and thus were used for plotting the PCA biplot. Overall, the first two PCs retained about 80% of the total variation in the dataset. The principal component 1 (PC1) contributing about 60% of the variation mainly served to separate the populations of PK and KS from the TBP site. Although, majority of the variables were associated with this component, however, the most contributing variables were the width of anther (AW), stamen width (STW), ovary length (OL), seed width (SW), style width (STLW), stamen length (STLL), and style length (SYL). Similarly, the principal component 2 (PC2) contributed about 20% of the variation in the dataset. Tepal length (TL), tepal width (TW), flower width (FW), flower number (FN), leaf size (LS), inflorescence length (LI), and length of rhizome (LR) were the most governing variables associated with this component (Figure 2).

### Anthesis and Anther Dehiscence

**FIGURE 3:**
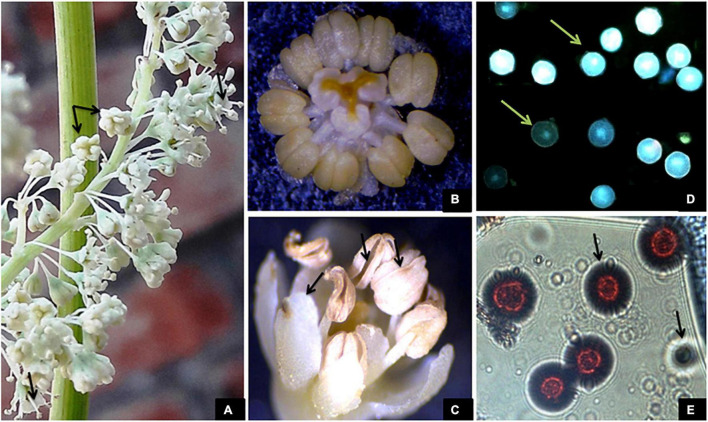
**(A)** Flower anthesis and outward movement of stamens taking anthers away from the stigma. **(B)** Hermaphrodite flower with 9 bithecous anthers and incurved stigmas. **(C)** Post-pollination incurved movement of stamens and petals and anther dehiscence. **(D,E)** Viable and non-viable pollen grains observed at 40× and 100× magnifications.

The flowers in the inflorescence do not show any particular fashion of opening. On an average, a flower took 2.4 h ± 20 min to open completely across all the study areas. The flowers began to open between 6:00 and 9:00 h, which continued till 14:15–16:30 h. Maximum flowers opened between 9:00 and 12:00 h (Table 4). None of the flowers open at night. A flower bears 9 stamens, each bearing a bithecous anther that faces toward the stigma (introrse). During bud condition, the filaments lie at an angle of 17° with respect to the ovary. However, elongation of stamens and their movement bring stamens at an angle of 70° with respect to the ovary at the time of anther dehiscence. The anthers are placed at an angle of 80° with respect to their filaments with the line of dehiscence facing toward the upper side. The anther dehiscence started at approximately 37.25 ± 3.3 h after flower anthesis. The anthers may or may not dehisce simultaneously. Dehiscence occurs along margins releasing a large number of pollen grains. The flowers may or may not shed all their pollen. Undehisced anthers wilt with their pollen inside and degenerate. The Tepals along with stamens then show inward movement and finally wither, which acts as a signal for fructification (Figures 3A–E).

**TABLE 4:** Pattern of flower anthesis and anther dehiscence.

Area	Anthesis	Time	Duration	Maximum	Completion	Anther dehiscence	Timing	Direction
TBP	May	6:00 a.m. ± 14 min	2.4 h ± 20 min	83% ± 5.8 at 09:30 a.m. ± 15 min	14:15 h ± 20 min	31.48 ± 1.58 h	A	80–120°
KS	June	07:15 a.m. ± 21 min	2.4 h ± 37 min	86% ± 7.7 at 12:00 noon ± 20 min	16:15 h ± 23 min	37.25 ± 3.3 h	A	70–110°
PK	June	09:00 a.m. ± 17 min	2.4 h ± 31 min	79% ± 5.1 at 11:15 a.m. 2.4 h ± 17 min	16: 30 h ± 17 min	38.55 ± 3.43 h	A	90–120°

*A, after anthesis.*

### Pollen Morphology, Viability, and the Pollen–Ovule Ratio

**FIGURE 4:**
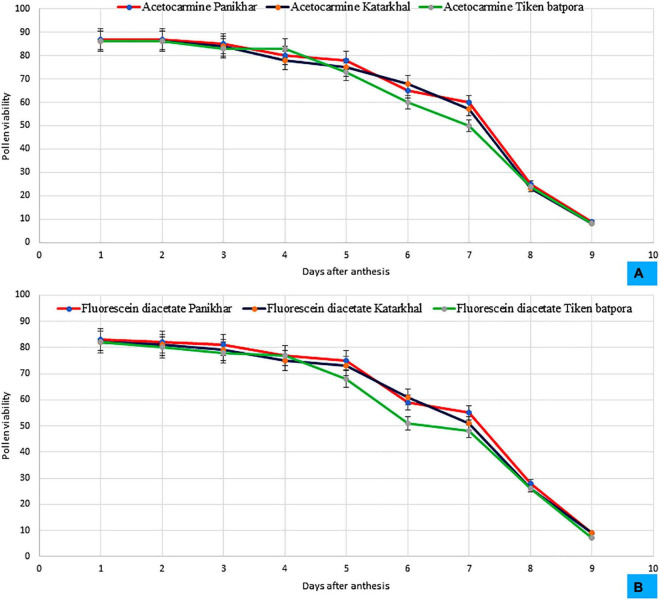
Pattern of the pollen viability at different populations observed in **(A)** 1% acetocarmine, **(B)** Fluorescein diacetate (FDA) (bars represent SD for *n* = 20 replicates).

During bud condition, the pollen is yellowish and become creamish white later on. Light and fluorescence microscopy revealed that the pollens are spherical and tricolpate. Exine bears spinulose sculpturing. The diameter of the pollen grain ranges between 2.57 ± 0.27 μm and 3.39 ± 0.22 μm. They are oval in shape. Pollen viability was observed at the time of anthesis, which showed a sharp decreasing trend and decreased to < 10% on day 9. Based on the daily average basis, pollen viability trend decreases between 09:00 and 21:00 h (09:00 > 12:00 > 15:00 > 18:00 > 21:00 h). The highest pollen viability as determined by the FDA test and 1% acetocarmine was reported at Panikhar (83 ± 4.2 and 87 ± 4.73%), followed by Tiken Batpora and Katarkhal (82 ± 4.98 and 86 ± 5.3%) (Figure 4). The pollen: ovule ratio was 7,000 ± 868:1, 6,086 ± 672:1, and 4,987 ± 478:1 per flower at Panikhar, Katarkhal, and Tiken Batpora, respectively.

### Stylar-Stigmatic Movement and Stigma Receptivity

The flowers are monocarpellary with a single pistil having three styles each terminating into a stigma. Fan-shaped stigmatic lobes are wet and fleshy. The styles are deflexed at an angle of 60° with respect to each other. Each stigmatic lobe has many small lobules. The average diameter of the stigma was highest at Panikhar, followed by Tiken Batpora and Katarkhal.

**FIGURE 5:**
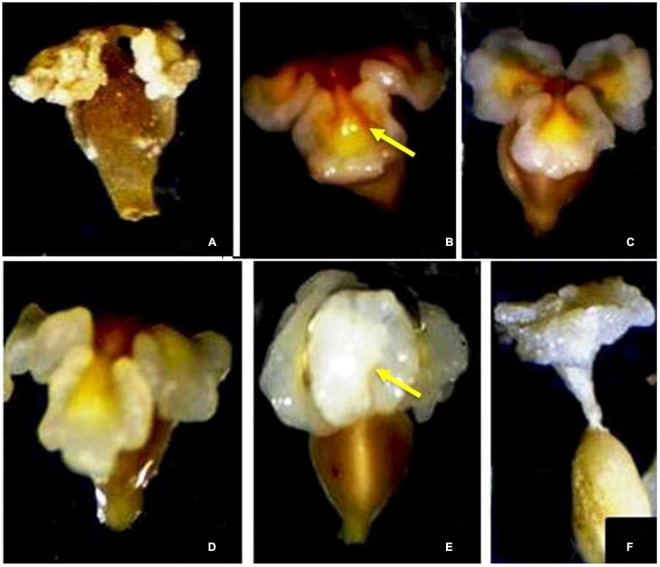
**(A–F)** Stigma-stylar movement in *Rheum webbianum.*

Prior to flower anthesis, the adaxial surfaces of the stigmas remain incurved at an angle of 360° with respect to the ovary. At anthesis, stigmatic lobes start an upward movement, and on days 3–6, the pollen receptive surface of each lobe comes to lie at 90°with respect to the ovary. By this time, almost 90% of the stigmas are pollinated. Once the pollen germination starts, these lobes swell and lose their receptivity. Furthermore, these pollinated stigmatic lobes move upward and come to lie at 180° with respect to the ovary, subsequently coalescing and forming a funnel-like structure through which pollen tubes move toward the ovary through the style. This movement completes in 6–7 days of anthesis (Figures 5A–F).

**FIGURE 6:**
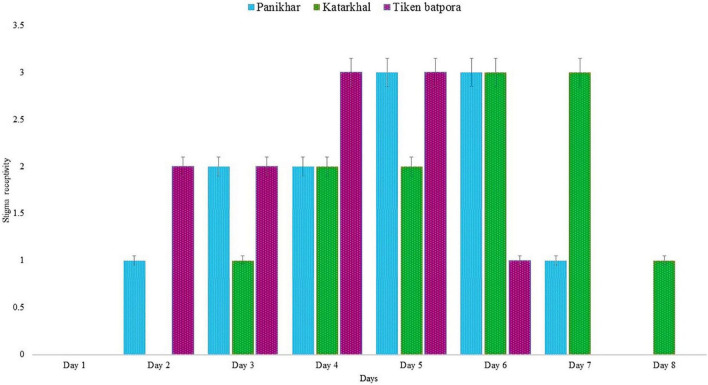
Stigma receptivity in *Rheum webbianum* where 0 represents no receptivity and 3.5 peak receptivity (bars represent SD for *n* = 20 replicates). Scale of 0–3.5 is based on the number of bubbles and pollen germinated on stigma.

The stigma receptivity was confirmed by pollen germination under a fluorescence microscope and bubbling in H_2_O_2_. The stigmas taken on day 1 of anthesis failed to show results and were thus designated non-receptive. The stigma receptivity was confirmed from days 2 to 8 of anthesis. Populations at Katarkhal show stigma receptivity on the third day of anthesis compared with the populations at Panikhar and Tiken Batpora, where stigma became receptive on the second day. The peak stigma receptivity (seen as the highest number of pollen germination and bubble formation) in Katarkhal was observed on days 6 and 7. In the population of Panikhar, the stigmas attained peak receptivity on days 5 and 6, while the populations at Tiken Batpora showed the highest receptivity on days 4 and 5. A decline in the stigma receptivity was seen from day 6, which finally ended on day 8. The complete pattern of stigma receptivity is explained in Figure 6.

### Fertilization

**FIGURE 7:**
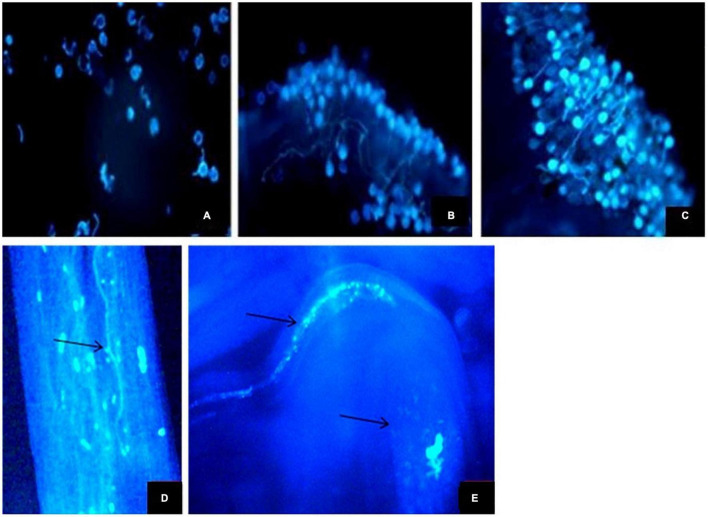
**| (A–E)** Fluorescent micrographs of pollen grains. **(A–C)** Pollen germination on the stigma surface **(D)** Pollen tubes in style. **(E)** Pollen tubes entering ovule.

A large number of pollen grains were found germinating on the stigmatic surface. The pollen tube takes about 2–3 h to reach the ovary. The stigmas were manually cross- and self-pollinated. The pollen tubes of both self- and cross-nature approximately took the same time to reach the ovules. After reaching a certain length, the growth of pollen tubes is blocked inside the style; however, a small number of pollen tubes traverse the style; and finally, a single pollen tube enters the ovule to initiate fertilization (Figures 7A–E).

### Fruit Formation and Reproductive Output

In *R. webbianum*, the fruit formation starts in May and ends in August. On an average, the plants bear 1,346 ± 303.9, 1,098 ± 241.4, and 784 ± 127.3 flowers of which 871 ± 94.6, 901 ± 121.4, and 591 ± 131.9 matured into fruits. The percent fruit set was 79.32, 78.62, 75.38%, and the percent seed set came to 91, 87, and 78%, respectively, at Panikhar, Katarkhal, and Tiken Batpora. A decrease in fruit set on the upper branches of the inflorescence was observed at TBP. One-third of fruits on such branches were either hollow or bore aborted seeds.

### Post-fertilization Changes

**FIGURE 8:**
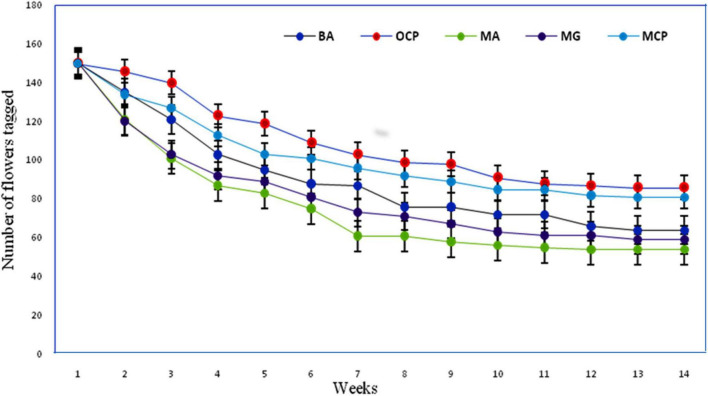
Percentage of fruit survival in *R. webbianum* after different manual treatments BA, bagged for autogamy; OCP, open cross-pollination; MA, manual autogamy; MG, manual geitonogamy; MCP, manual cross-pollination.

The post-fertilization changes were observed through 14-week long period and the entire developmental processes were taken into consideration. Withering of the flowers within the first 2 weeks was recognized as a signal for the failure of fertilization. The fruits matured within a period of 98 ± 14.4 days (from pollination up to seed maturation). Manually self-pollinated flowers (autogamy and geitonogamy) showed rapid abscission during the first 5 weeks. The rest of the flowers showed progressive development with less abscission till the complete maturation of the fruits. Open and manually cross-pollinated and bagged flowers show a lesser abscission rate during their early post-fertilization development and a steady abscission rate until last week (Figure 8).

### Seed Germination and Seed Survival

**FIGURE 9:**
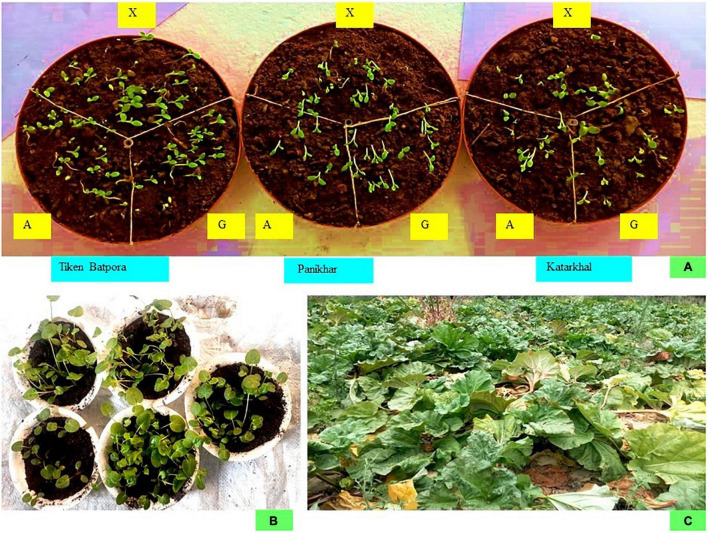
Seed germination and seedling transplantation. **(A)** Seed germination results of the seeds obtained from manual treatments X, xenogamy; G, geitonogamy; A, autogamy. **(B)** Root hardening. **(C)** Transplanting the plants into field conditions.

The seeds obtained from different pollination treatments showed a considerable change in their mean weight. The maximum weight was obtained after 78 h of imbibition, after which a constant weight was obtained for all the samples. After sowing, the seeds started to germinate on the 7th day and continued up to 16th day. The highest germination was reported for seeds obtained from xenogamy followed by geitonogamy and autogamy in all the populations. The highest mean germination percentage and seedling survival was reported for Tiken Batpora, followed by Panikhar and Katarkhal (Table 5 and Figures 9A–C).

**TABLE 5:** Seed germination and seedling survival in *Rheum webbianum* (*n* = 25).

Treatment	Dry weight (milligrams)			Maximum weight after imbibition (milligrams)			Germination (percentage)			Seedling survival (percentage)		
	PK	KS	TBP	PK	KS	TBP	PK	KS	TBP	PK	KS	TBP
Xenogamy	32 ± 0.29	29 ± 0.23	35 ± 0.48	75 ± 5.5	71 ± 4.4	78 ± 6.1	94 ± 8.4	91 ± 6.6	95 ± 4	82 ± 3	79 ± 3	84 ± 5.2
Geitonogamy	29 ± 0.25	28 ± 0.22	31 ± 0.54	62 ± 4.7	59 ± 4.1	66 ± 5.5	90 ± 7.8	89 ± 4.3	92 ± 4	79 ± 4	75 ± 3	81 ± 4.9
Autogamy	27 ± 0.25	25 ± 0.18	28 ± 0.51	65 ± 4.9	61 ± 4.6	69 ± 4.9	88 ± 6	88 ± 4.4	89 ± 3.5	76 ± 4	72 ± 3	78 ± 4.2

### Breeding System

Different experiments on breeding behavior revealed that it is self-compatible and cross-fertile. None of the emasculated and bagged flowers set fruit, indicating that no apomixis or agamospermy occurs in *R. webbianum*. Manual self-pollination confirms autogamy as well as geitonogamy. The fruit and seed set of cross-pollinated flowers (CP) was significantly higher than that of self-pollinated flowers (SP) (autogamy and geitonogamy). The highest fruit set was observed for open-pollinated flowers [open pollination (OP): flower (FR) = 103, percent fruit set (%FS) = 68.66, percent seed set (%SS) = 86 *P* < 0.001- emasculated and manually cross pollinated (EMCP): FR = 92,% FS = 61.33,%SS = 81 *P* < 0.001] compared with self-pollinated flowers [unemasculated flowers bagged (UEB): FR = 59,%FS = 39.33,%SS = 72.8 *P* < 0.001- manual geitonogamy (MG): FR = 56,%FS = 37.33,%SS = 69 *P* < 0.001- manual autogamy (MA): FR = 52,%FS = 34.66,%SS = 63.64 *P* < 0.001]. The reproductive efficacy index as determined for the plant was quite low at 0.71, which ranged between 0.5 and 0.75 at the 95% CI. Detailed results of the breeding system are explained in Table 6. The results of the two-way ANOVA indicate that there is a significant effect of treatment on both the fruit and seed development, but the effect of site turned out to be non-significant.

**TABLE 6:** Seed set in *Rheum webbianum* following different treatments.

Treatments	Plants	Flowers	Fruits	% Fruit set	Seeds	% seed set
Emasculated and bagged (apomixis)	6	150 ± 5.77	0	0	0	0
Un emasculated flowers bagged (bud conditions)	6	150 ± 13.81	59	39.33%	43	72.8
Manual autogamy	6	150 ± 5.77	52	34.66%	33	63.64
Manual getinogamy	6	150 ± 5.37	56	37.33%	39	69.64
Open pollination	6	150 ± 5. 52	103	68.66%	86	86
Emasculated and manually cross pollinated	6	150 ± 5.16	92	61.33%	81	81

### Outcrossing Index, Index of Self-Incompatibility, and Inbreeding Depression

The outcrossing index of Cruden was maximum, strongly favoring the outcrossing nature of the species. A maximum score of 5 was found for all the populations. The ratio of percent seed set obtained on manual self-: cross-pollination treatment is quite high (0.7) from its threshold level (≤ 0.2), which revealed the self-compatible nature of this plant species. A significant level of inbreeding depression (*P* < 0.01) was reported for self-pollinated fruits. The fruits obtained on xenogamy were heaviest, followed by geitonogamy and autogamy (Table 5).

### Pollination Mechanism

The flowers of *R. webbianum* are both entomo- and anemophilous. The plants are both self-compatible and cross-fertile. Some populations had only a few distantly placed individuals (approximately 100 m). Such populations undergo either auto- or geitonogamy through the mediation of strong winds blowing from north to south at Katarkhal (3,474 m.a.s.l.) and from north to the westward at Panikhar (3,227 m.a.s.l.). At Tiken Batpora (1,992 m.a.s.l.), the pollination was facilitated only by insects (entomophily). It was confirmed through a hanging slide experiment that at TBP, no pollen was observed on the hung slides around the plants, whereas the insects collected were laden with conspecific pollen on their body parts. Airborne pollen that was caught on Meyers albumin smeared on hanging slides confirmed the role of wind in facilitating cross-pollination in the Panikhar and Katarkhal populations. The pollination occurs both during the day as well as at night. Sixty-six of the 100 flowers unbagged during the day matured into fruits, compared with 37 of the 100 flowers that were unbagged only at night.

### Insect Visitors and Their Pollination Indices

A total of 16 different insect visitors were observed, belonging to four orders (Hymenoptera, Diptera, Lepidoptera, and Thysanoptera), nine families, and 11 genera. Hymenoptera was observed as the most dominant order, with 12 insect visitors. Pollinator abundance and richness were highest at Tiken Batpora, followed by Panikhar and Katarkhal. Based on different pollination indices, *Apis cerana* and *Apis mellifera* at Tiken Batpora, *Bombus lucorum*, and *Lasioglossum* sp. at Katarkhal and *Bombus asiaticus*, *Bombus ferganicus*, and *Aphelinus* sp. at Panikhar were classified as the dominant pollinators. Comparative pollination indices of different pollinators are given in Table 7. Small pollen load was observed on the body parts of *Lasioglossum* sp., *Musa domestica, Vanessa cardui, Xylocopa* sp., *Thrips*, and *Xylocopa valga* and hence were considered inefficient pollinators or robbers. One-way ANOVA showed that there was a significant difference in the pollen load on body parts for different pollinators. Thorax and abdomen showed the highest number of pollen grains followed by wings and head (Table 8).

**TABLE 7:** Pollination indices of different visitors across different study areas.

Site	Pollinator	Foraging behavior	Insect visiting efficiency	Index of visitation rate
TBP	*Apis cerana*	5.7 ± 0.91	0.159 ± 0.07	189 ± 23.3
	*Apis mellifera*	4.94 ± 0.79	0.113 ± 0.09	204 ± 17.7
	*Allograpta* sp.	2.7 ± 0.43	0.068 ± 0.03	35.9 ± 8.5
	*Musa domestica*	1.17 ± 0.37	0.02 ± 0.01	24.85 ± 7.7
	*Camponotus* sp.	8.19 ± 1.15	0.15 ± 0.11	478.9 ± 76.16
	*Eupeodes corollae*	3.61 ± 1.19	0.143 ± 0.11	47.07 ± 12.02
	*Vanessa cardui*	1.17 ± 0.39	0.09 ± 0.068	7.71 ± 2.2
	*Vespula* sp.	0.73 ± 0.22	0.05 ± 0.026	5 ± 4.5
KS	*Lasioglossum* sp.	4.17 ± 1.19	0.109 ± 0.09	27.79 ± 4.4
	*Bombus lucorum*	5.17 ± 2.2	0.227 ± 0.09	204 ± 22.72
	*Eupeodes corollae*	5.05 ± 0.91	0.339 ± 0.12	317 ± 17.7
	*Xylocopa valga*	0.55 ± 0.21	0.073 ± 0.021	6.6 ± 2.54
	*Eupeodes corollae*	1.23 ± 0.29	0.03 ± 0.009	10.17 ± 3.3
	*Musa domestica*	1.59 ± 0.12	0.05 ± 0.01	36.06 ± 7.72
PK	*Aphelinus* sp.	4.97 ± 0.98	0.227 ± 0.063	187 ± 13.3
	*Aphidius* sp.	4.12 ± 0.92	0.161 ± 0.097	89 ± 9.9
	*Bombus asiaticus*	4.97 ± 0.28	0.193 ± 0.11	119 ± 15.5
	*Bombus ferganicus*	7.73 ± 2.28	0.229 ± 0.085	172 ± 17.34
	*Bombus melanurus*	6.9 ± 2.16	0.277 ± 0.067	138 ± 26.9
	*Xylocopa* sp.	1.05 ± 0.51	0.093 ± 0.05	14.4 ± 4.54

**TABLE 8:** Pollen load on different body parts of insect visitors.

Insect visitor	Order	Family	E.P	Pollen load				
				Head	Thorax	Abdomen	Wings	Legs
*Aphelinus* sp.	Hymenoptera	Aphelinidae	M	11 ± 5.5	247 ± 121.14	119 ± 79.83	27 ± 14.09	15 ± 4.4
*Aphidius* sp.	Hymenoptera	Braconidae	M	9.9 ± 5.8	217.7 ± 137.7	161.09 ± 92.09	41.69 ± 21.01	17 ± 7.9
*Apis cerana*	Hymenoptera	Apidae	H	121 ± 47.21	479.3 ± 192.9	224.1 ± 132.7	92 ± 37.91	21 ± 11.9
*Apis mellifera*	Hymenoptera	Apidae	H	127 ± 55.05	512 ± 292.5	314 ± 142	63 ± 23.09	29 ± 17.07
*Bombus asiaticus*	Hymenoptera	Apidae	H	87.5 ± 23.9	313.7 ± 107	198 ± 72.07	58.09 ± 22.19	37.17 ± 13.9
*Bombus ferganicus*	Hymenoptera	Apidae	H	83 ± 27.07	273.8 ± 47.8	149 ± 23.06	41 ± 13.01	39.09 ± 13
*Bombu s lucorum*	Hymenoptera	Apidae	H	53.8 ± 13.9	267.7 ± 71. 9	211 ± 51.81	53.3 ± 9.9	27.07 ± 11.3
*Bombus melanurus*	Hymenoptera	Apidae	H	83 ± 21.09	277.9 ± 39.9	109.9 ± 17.7	32 ± 9	17 ± 8.3
*Camponotus* sp.	Hymenoptera	Formicidae	H	27 ± 9.09	72.7 ± 21.9	51.13 ± 12.02	14.7 ± 5	9.5 ± 7.7
*Eupeodes corollae*	Diptera	Syrphidae	M	19 ± 5.9	47.09 ± 13.03	29.12 ± 6.6	–	–
*Lasioglossum* sp.	Hymenoptera	Halictidae	M	7.07 ± 3.9	31.17 ± 10.9	39.89 ± 12.02	–	–
*Musa domestica*	Diptera	Muscidae	L	–	19 ± 4.7	17.07 ± 4.3	–	13 ± 3
*Vanessa cardui*	Lepidoptera	Nymphalidae	L	–	–	19.09 ± 4.2	–	21.09 ± 11
*Xylocopa* sp.	Hymenoptera	Apidae	L	–	47 ± 17.07	14 ± 5.1	–	–
*Xylocopa valga*	Hymenoptera	Apidae	L	–	53.8 ± 16.06	23 ± 6	–	–
*Thrips*	Thysanoptera	Thripidae	L	16.06 ± 4.9	23 ± 5.9	21.09	7.14 ± 2.21	5.5 ± 2.2

*EP, Effective pollinator; H, High; L, Low; M, Moderate; Ab, Abdomen.*

### Pollination Efficiency and Behavior of Pollinators

The pollination efficiency of the pollinators was evaluated on the basis of pollen load attached to their body parts and its subsequent deposition on the stigma of another flower. The natural pollination efficiency ranged between 39 ± 21.1 and 421 ± 71.90 pollen grains per stigma, which was enough to fertilize a single ovule in the flower. The compact arrangement of flowers in terminal panicle inflorescences provides a platform to pollinators for easy landing. Depending on the size of the pollinators, landing requires 3–8 ± 2.1 flowers. On average, 7 out of 10 pollinators landed in the middle portion of the inflorescence from which they were seen either moving upward or downward. A single visit of a pollinator resulted in gnawing of 3–12 ± 2.2 flowers. The average time spent by a pollinator for doing so ranged between 3–42 ± 2.2 s.

While foraging nectar, the pollinators received pollen from dehisced anthers and subsequently on a visit to another flower (from the same or a different plant), such pollen was transferred to the stigma. The elevated position of anthers at dehiscence results in deposition of the highest pollen load on the cephalothoraxic region, followed by the abdomen, legs, and wings of such insects. The pollen attached to the proboscis of some pollinators leads to pollination of different flowers. *Apis* spp. and *Bombus* spp. were the pollinators for more behavioral observations. These were the most frequent visitors, moving very quickly between the plants in the population and among the branches of inflorescence. *Apis* spp. crawled over the flowers and inserted their proboscises into the base of the ovary. On some of the occasions, these pollinators were seen moving up the inflorescences first and then down, visiting previously skipped flowers. Such pollinators never revisited the flowers that were gnawed during the particular visits to an inflorescence. While collecting the nectar, the lateral and ventral sides of the abdomen of such pollinators get brushed against anthers, resulting in deposition of a copious amount of pollen grains on their body parts. Bumblebees were seen landing directly on the stigma of the flower. While making an effort to lick the nectar, their posterior body parts touch the dehisced anthers. *Eupeodes corollae, Aphidius* sp., and *Aphelinus* sp. keep their abdomens elevated and inclined while resting on flower tepals. *Lasioglossum* sp., *Musa domestica, Allograpta* sp., *Vanessa cardui, Xylocopa* sp., and *Xylocopa valga* fly over and around the inflorescences before a brief stay on the flowers, stretching their mouthparts through petal gaps to reach the nectary located at the base of the perianth. *Stomoxy* sp., and *Vespula* sp., crawled in a circular fashion around the margins of the stigmatic lobes before landing on the adaxial surface of the stigma.

### Insect Visitation *vis-a-vis* Environmental Factors

**FIGURE 10:**
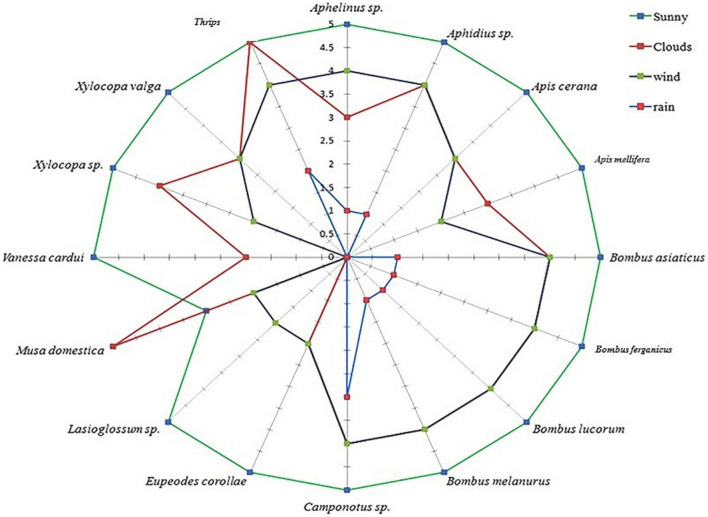
Change in the pollinator visiting frequency during different weather conditions.

External environmental factors, such as wind, clouds, rain, and temperature, showed a significant influence on the visiting behavior of insect pollinators. However, ants, aphids, thrips, beetles, and bugs were the least affected. On normal sunny days, the insects were observed visiting early in the morning and continued throughout the day. The frequency of insect visitation increased with increasing temperature and illumination. The peak insect visitation was reported between 12:00 and 14:00 h, which declined thereafter. The highest visitation frequency of pollinators was reported on bright sunny days, which declined because of clouds, wind, and rain (Figure 10).

## Discussion

### Floral and Reproductive Phenology

The phenology of a plant species not only deals with the vegetative and reproductive phases corresponding to the climate and seasonal changes of a particular area but also determines the degree of reproductive synchrony with other plant species (Rathcke and Lacey, 1985). A key tool for the plant management is to have the information on floral biology and estimation of reproductivity and regeneration (Mulik and Bhosale, 1989). During the present study on *R. webbianum*, it was observed that phenophases of the plant species showed a significant variation across the selected populations. Elevated temperatures accelerate plant development processes and as such advance the different phenophases (Gordo and Sanz, 2010; Bjorkman et al., 2015; Moore and Lauenroth, 2017; Dorji et al., 2020). At lower altitudes, *R. webbianum* showed early germination, which could be attributed to the difference in temperature as well as available moisture as reported by workers in other plant species (Tao et al., 2008; Julien and Sobrino, 2009). Populations at Katarkhal showed lowest leaf size and number, length and width of the petiole, length and thickness of the rhizome, and inflorescence size and width. These results can be related to the findings of Siddique et al. (1997), Bresson et al. (2011), and Yaqoob and Nawchoo (2015) who also reported a reduction in the phenological characters of plants with an increase in elevations. According to Korner (2003) and Baret et al. (2004), reduction in the morphometric characters is the adaptation of alpine plants that may result due to slow growth, which allows the alpine plants to use the resources more efficiently. Smaller inflorescences at higher altitudes help plants to avoid the detrimental consequences of strong and gusty winds (Korner and Cochrane, 1983; Yaqoob and Nawchoo, 2015).

### Flower Anthesis

Anthesis is a vital event in the morphogenesis and overall development of the flower (Smitha and Thondaiman, 2016; Kumari et al., 2020). The bud dormancy of many plant species is broken when forced to experience high temperatures (Rohde and Bhalerao, 2007). With the increase in temperature, the flower anthesis hastened in *R. webbianum*, as cracks on flower buds appeared with early sunshine, resulting in the separation of perianth lobes. These findings are supported by the results of De Hertogh and Le Nard (1993) and Noy-Porat et al. (2009) who reported the favorable role of high temperature in flower anthesis in *Narcissus* and *Tulipa* inhabiting temperate to subalpine conditions.

### Pollen Biology, Stigma Receptivity, and Pollen Pistil Interaction

The pollen viability and the efficiency of pollen transfer are important steps indicating the reproductive success of a plant species (Khan et al., 2021). A reduction in pollen viability is a common trait in angiosperms growing under changing weather conditions (Porch and Jahn, 2001; Fang et al., 2010). A slight decrease in the pollen viability at populations of Katarkhal as compared with that of Panikhar can be attributed to the climatic stress induced due to changing weather conditions during the reproductive phase of *R. webbianum*. Pollen viability declined with the age of flower and such findings are in line with the studies of Mercado et al. (1994) and Rodriguez-Riano and Dafni (2007), who reported a steady decline in pollen viability with the aging of flowers.

For the successful initiation of pollen pistil interaction, pollen viability and stigma receptivity are important parameters (Verma et al., 2004; Khajuria et al., 2011). Stigma receptivity can be useful in determining the optimum time to perform manual pollination besides being helpful in describing pollen/stigma incompatibility (Dafni, 1992). Details of the stigma receptivity in the plant species are described by a limited number of studies (Shivanna, 2003), as there are no satisfactory precise methods to determine the stigma receptivity. The presence of certain enzymes, such as acid phosphatases, peroxidases, and esterases, mark the receptivity of stigmas (Dafni, 1992; Dafni and Maués, 1998) but this may not be true for all the cases, as in some species, these enzymes are secreted before the stigmas get receptive (Shivanna and Sastri, 1981). In these cases, manual pollination treatments could only be a definite method to determine the stigma receptivity (Shivanna, 2003). In case of *R. webbianum*, the pattern of pollen germination on stigma and bubble formation in H_2_O_2_ showed similar results. Moreover, extended stigma receptivity was seen in *R. webbianum*, which assures fertilization and reproductive success (Castro et al., 2008). This time frame appears to be an approach to ensure reproductive output in this species growing under extreme climatic conditions.

### Dichogamy and Stigma Movement, a Contrivance for Cross-Pollination

Dichogamy is believed to have progressed to contribute toward outbreeding that limits pollen and stigma interference in the same flower or within the plant (Bertin and Newman, 1993; Barrett, 2002). Delayed selfing through stylar movement (Verma et al., 2004; Ruan et al., 2009) and flexistyly (Li et al., 2001) are some mechanisms that promote outcrossing in some plant species. Incurved stigmas at anthesis in *R. webbianum* seem to limit the chances of self-pollination in this species. Dichogamy (protandry), reverse herkogamy, and stylar-stigmatic movements strongly promote outcrossing in this species. Cross-pollination not only maintains certain levels of heterozygosity in the populations but also prevents certain levels of inbreeding depression (Hu et al., 2011; Li et al., 2019).

### Breeding System

The breeding system plays an important role in the generation of variation and hence in the evolution of a species (Grant, 1971). The pollen-ovule ratio (P/O) is the best indicator of a species’ breeding pattern (Gallardo et al., 1994). The greater pollen ovule ratio in *R. webbianum* indicates that xenogamy and facultative xenogamy are the most operative modes of pollination exhibited by this plant species. The same was confirmed by undertaking bagging experiments. *R. webbianum* practiced facultative xenogamy under pollinator limiting conditions. This strategy of mixed mating appears to be a survival approach by *R. webbianum* under extreme climatic conditions. However, its stigmatic movement effectively prevents self-pollination and acts as a contrivance for cross-pollination indicating that the breeding system of *R. webbianum* is in the process of evolving from selfing to outcrossing. This finding is supported by the results of Ganie et al. (2017), who also reported a similar mechanism in *R. webbianum*. Although cross-pollination contributes to heterozygosity, self-pollination under limiting conditions helps the plant to set seeds and to survive challenging environmental conditions. The same kind of observations has been reported by Huang et al. (2006) and Khajuria (2013) for other plant species, in which plants shift to self-pollination mode under limiting conditions.

### Pollination Ecology

The mutualism between the plants and the pollinators depends on the exchange of food and efficient vectoring of sexual reproduction for plants. During the course of evolution, these interactions have been modified by a variety of factors, which include both biotic as well as abiotic constraints. While a large group of pollinators is generalists (Hingston and McQuilan, 2000; Ollerton, 2014), there are some specialists that have evolved throughout the course of evolutionary history (Proctor et al., 1996; Endress and Bruyns, 2000). Bumblebees are believed to have coevolved with the climatic conditions at alpine zones and can happily pollinate the plants growing up to the altitude of 9,000 m.a.s.l. (Dillon and Dudley, 2014). Preferentially, bumblebees constitute the group of specialist pollinators for *R. webbianum*, which is supported by various pollination indices. This analysis is equally supported by the findings of Pradervand et al. (2014), who also found such pollinator groups to be dominant in the other alpine regions.

The floral morphology is of greater importance, as it determines the pollination behavior of different plant species (Vikas, 2011; Yamasaki and Sakai, 2013). Leafless terminal inflorescences, small perianth, exposed stigmatic surface, raised anthers, and lightweight pollen are anemophilous floral features in *R. webbianum*. Features like dichogamy (protandry), reverse herkogamy, incurved stigmas, delicate fragrance, nectar availability, and dense and showy inflorescences favor insect pollination. The importance of such traits for attracting pollinators has also been reported in other plant species (Dafni, 1992; Mayer et al., 2011; Yamasaki and Sakai, 2013). The data generated in the case of *R. webbianum* prompted us to design an *ex situ* conservation strategy for the management of this important but threatened high-value medicinal plant.

### Conservation Strategy

The present study on reproductive biology featuring phenology, floral traits, pollination, and breeding behavior has provided mechanistic insights into the life history pattern of *R. webbianum*, a vulnerable medicinal plant of North-West Himalaya. The species faces anthropogenic stress in nature and disjunct population distribution makes it more susceptible to exploitation. Since the species depends upon specialist pollinators, any climatic change affecting the pollinator availability and abundance will be affecting its survival too. Low seed germinability under *in situ* conditions is also a cause of concern. On the basis of the information generated so, an effective conservation program for the species will be comprised of both *in situ* and *ex situ* approaches. Since the species shows habitat specificity, all its habitats need to be protected. The exploitation of the species needs to be stopped through the intervention of forest department as well as other law enforcing agencies. The *in situ* populations need to be protected from grazing and trampling. It would save the already sparse populations from damage and further destruction. *Ex situ* conservation and seed collection should then be carried out to provide for its future recovery. Restoration of natural populations using *ex situ* raised seedlings and saplings will also be contributing toward the recovery of this very important medicinal plant.

## Conclusion

The present study revealed that *R. webbianum* grows in disjunct populations at different elevations and a wide range of habitats in Kashmir Himalaya. The species was found to inhabit dry and exposed mountain peaks, moist and bushy habitats, and plains with loose textured soil. Variability in the morphological traits of this species was observed across different altitudinal gradients, which reflect its phenotypic plasticity and adaptability under different climatic conditions. The plant bears hermaphrodite, dichogamous flowers exhibiting reverse herkogamy and stylar–stigmatic movements to promote outcrossing. It is both self-compatible as well as cross-fertile and ambophilous. The dominance of Hymenopteran pollinators at all the study sites indicates toward the role of specific pollinators in pollination and its reproduction. The species practice mixed mating but manual pollination experiments point toward its outbreeding nature. The information generated thereof reveals that the species exploit both phenotypic as well as reproductive plasticity to survive under limiting and stressful conditions of nature. The information generated so is of great significance and will go a long way in designing the effective strategies for its cultivation, conservation, and sustainable use.

## Data Availability Statement

The original contributions presented in the study are included in the article/supplementary material, further inquiries can be directed to the corresponding author/s.

## Author Contributions

SV contributed to conceptualization, validation, investigation, data curation, visualization, and supervision. IW and SV contributed to methodology, formal analysis, and writing original draft. IW and PA contributed to software. SV and PA contributed to resources and project administration. SV, IW, and PA contributed to writing, reviewing, and editing. HE-S and MH contributed to funding acquisition. All authors have read and agreed to the published version of the manuscript.

## Conflict of Interest

The authors declare that the research was conducted in the absence of any commercial or financial relationships that could be construed as a potential conflict of interest.

## Publisher’s Note

All claims expressed in this article are solely those of the authors and do not necessarily represent those of their affiliated organizations, or those of the publisher, the editors and the reviewers. Any product that may be evaluated in this article, or claim that may be made by its manufacturer, is not guaranteed or endorsed by the publisher.

## References

[B1] Abe I. Seki T. Noguchi H. Kashwada Y. (2000). Galloyl esters from rhubarb are potential inhibitors of squaleene epoxidase, a key enzyme in cholestrol biosynthesis. *Planta Med.* 66 753–756. 10.1055/s-2000-9781 11199136

[B2] Agarwal S. K. Singh S. S. Lakshmi V. (2001). Chemistry and Pharmacology of Rhubarb (Rheum species)- A review. *J. Scient. Ind. Res.* 60 1–9.

[B3] Anderson G. J. Johonson S. D. Neal P. R. Bernardello G. (2002). Reproductive biology and plant systematics: the growth of symbiotic association. *Taxon* 51 637–653. 10.2307/3647326

[B4] Ashman T. L. (2003). Constraints on the evolution of males and sexual dimorphism: field estimates of genetic architecture of reproductive traits in three populations of gynodioecious *Fragaria virginiana*. *Evolution* 57 2012–2025. 10.1111/j.0014-3820.2003.tb00381.x 14575323

[B5] Baig B. A. Ramamoorthy D. Wani B. A. (2014). Population status and conservation prioritization of some threatened medicinal plants of Kashmir Himalaya. *Int. J. Appl. Biol. Pharm. Technol.* 4 21–29.

[B6] Bareke T. (2018). Biology of seed development and germination physiology. *Adv. Plants Agric. Res.* 8 336-346. 10.15406/apar.2018.08.00335

[B7] Baret S. Maurice S. Le Bourgeois T. Strasberg D. (2004). Altitudinal variation in fertility and vegetative growth in the invasive plant *Rubus alceifolius* (Rosaceae), on Reunion Island. *Plant Ecol.* 172 265–273. 10.1023/B:VEGE.0000026345.67250.d2

[B8] Barman C. Singh V. C. Das S. Tandon R. (2018). Floral contrivances and specialized pollination mechanism confer strong influence to elicit mixed-mating in *Wrightia tomentosa* (*Apocynaceae*). *Plant Biol.* 20 546–554. 10.1111/plb.12690 29330901

[B9] Barrett S. C. H. (2002). The evolution of plant sexual diversity. *Nat. Genet.* 3 274–284. 10.1038/nrg776 11967552

[B10] Becker U. Colling G. Dostal P. Jakobsson A. Matthies D. (2006). Local adaptation in the monocarpic perennial *Carlina vulgaris* at different spatial scales across Europe. *Oecologia* 150 506–518. 10.1007/s00442-006-0534-9 16955286

[B11] Bentos T. V. Rita C. G. Mesquita G. Williamson B. (2008). Reproductive Phenology of Central Amazon Pioneer Trees. *Trop. Conserv. Sci.* 1 186–203. 10.1177/194008290800100303

[B12] Bertin R. I. Newman C. N. (1993). Dichogamy in angiosperms. *Bot. Rev.* 59 112–150. 10.1007/BF02856676

[B13] Bjorkman A. D. Elmendorf S. C. Beamish A. L. Vellend M. Henry G. H. R. (2015). Contrasting effects of warming and increased snowfall on Arctic tundra plant phenology over the past two decades Glob. *Chang. Biol.* 21 4651–4661. 10.1111/gcb.13051 26216538

[B14] Brauner S. Gottlieb L. D. (1987). A self-compatible plant of *Stephanomeria exigua* subsp. *coronaria* (Asteraceae) and its relevance to the origin of its self-pollinating derivative *S. malheurensis*. *Syst. Bot.* 12 299–304. 10.2307/2419325

[B15] Bresson C. C. Vitasse Y. Kremer A. Delzon S. (2011). To what extent is altitudinal variation of functional traits driven by genetic adaptation in European oak and beech? *Tree Physiol.* 31 1164–1174. 10.1093/treephys/tpr084 21908436

[B16] CAMP (2003). *Conservation Assessment and Management Prioritization Workshop for Medicinal Plants of Northwest Himalayan states of Jammu & Kashmir, Himachal Pradesh and Uttaranchal.* Bangalore: Foundation for Revitalisation of Local Health Traditions (FRLHT).

[B17] Castano A. M. Vilà M. Sanchez J. O. (2014). Pollination ecology of a plant in its native and introduced areas. *Acta Oecol.* 56 1–9. 10.1016/j.actao.2014.01.001

[B18] Castro S. Silveira P. Navarro L. (2008). How flower biology and breeding system affect the reproductive success of the narrow endemic *Polygala vayredae* Costa (Polygalaceae). *Bot. J. Linn. Soc.* 157 67–81. 10.1111/j.1095-8339.2008.00784.x

[B19] Chaurasia O. P. Ballah B. (2009). Medicinal Plants of cold desert Ladakh used in treatment of stomach disorders. *Indian J. Trad. Know.* 82 185–189.

[B20] Dafni A. (1992). *Pollination Ecology.* (Oxford: Oxford University Press), 1–57.

[B21] Dafni A. Maués M. M. (1998). A rapid and simple procedure to determine stigma receptivity. *Sex. Plant Reprod.* 11 177–180. 10.1007/s004970050138

[B22] De Hertogh A. A. Le Nard M. (1993). *The Physiology of Flower Bulbs: A Comprehensive Treatise on the Physiology and Utilization of Ornamental Flowering Bulbous and Tuberous Plants.* Amsterdam: Elsevier Science Publishers, 617–682.

[B23] Dillon M. E. Dudley R. (2014). Surpassing Mt. Everest: extreme flight performance of alpine bumble-bees. *Biol. Lett*. 10:20130922. 10.1098/rsbl.2013.0922 24501268 PMC3949368

[B24] Dorji T. Hopping K. A. Meng F. Wang S. Jiang L. Klein J. A. (2020). Impacts of climate change on flowering phenology and production in alpine plants: the importance of end of flowering. *Agric. Ecosyst. Environ.* 291:106795. 10.1016/j.agee.2019.106795

[B25] Endress M. E. Bruyns P. V. (2000). A revised classification of the *Apocynaceae*. *Bot. Rev.* 66 1–56. 10.1007/bf02857781

[B26] Evans J. D. Pettis J. S. Hood W. M. Shimanuki H. (2003). Tracking an invasive honey bee pest: mitochondrial DNA variation in Noth American small hive beetles. *Apidologie* 34 103–109. 10.1051/apido:2003004

[B27] Fang X. Turner N. C. Yan G. Li F. Siddique K. H. M. (2010). Flower numbers, pod production, pollen viability, and pistil function are reduced and flower and pod abortion increased in chickpea (*Cicer arietinum* L.) under terminal drought. *J. Exp. Bot.* 61 335–345. 10.1093/jxb/erp307 19854801 PMC2803204

[B28] Gallardo R. Dominguez E. Munoz J. M. (1994). Pollen ovule ratio, pollen size, and breeding system in Astragalus (Fabaceae) subgenus Epiglotis. A pollen and seed allocation approach. *Am. J. Bot.* 81 1611–1619. 10.1002/j.1537-2197.1994.tb11473.x

[B29] Gan X. Cao L. Zhang X. Li H. (2013). Floral biology, breeding system and pollination ecology of an endangered tree *Tetracentron sinense* Oliv. (Trochodendraceae). *Bot. Stud.* 54:50. 10.1186/1999-3110-54-50 28510885 PMC5430371

[B30] Ganie A. H. Tali B. A. Khuroo A. A. Nawchoo I. A. Rather A. M. (2014). *Rheum spiciforme* Royle (Polygonaceae): a new record to the flora of Kashmir Valley, India. *Natl. Acad. Sci. Lett.* 37 561–565. 10.1007/s40009-014-0279-7

[B31] Ganie A. H. Tali B. A. Reshi Z. A. Nawchoo I. A. (2017). Stigmatic Movement Promotes Cross Pollination in *Rheum webbianum* Royle: an Important Endemic Medicinal Plant of Kashmir Himalaya. *Natl. Acad. Sci. Lett.* 40 435–438. 10.1007/s40009-017-0579-9

[B32] Gopalakrishnan K. K. Thomas T. D. (2014). Reproductive biology of *Pittosporum* dasycaulon Miq., (Family Pittosporaceae) a rare medicinal tree endemic to Western Ghats. *Bot. Stud.* 55:15. 10.1186/1999-3110-55-15 28510916 PMC5432743

[B33] Gordo O. Sanz J. J. (2010). Impact of climate change on plant phenology in Mediterranean ecosystems. *Glob. Change Biol.* 16 1082–1106. 10.1111/j.1365-2486.2009.02084.x

[B34] Grant V. (1971). *Plant Speciation.* New York, NY: Columbia University Press.

[B35] Hingston A. B. McQuilan P. B. (2000). Are pollination syndromes useful predictors of floral visitors in Tasmania? *Aust. Ecol.* 25 600–609. 10.1111/j.1442-9993.2000.tb00065.x

[B36] Hu W. Q. Lu H. Liu W. Yuan J. X. Zhang D. (2011). Paternity identification and genetic structure analysis of the wild population in *Paeonia lactiflora* Pallas (Paeoniaceae). *Acta Hortic. Sin.* 38 503–511.

[B38] Huang Y. Zhang C. Q. Blackmore S. Li D.-Z. Wu Z.-K. (2006). A preliminary study on pollination biology of *Omphalogramma souliei* Franch. (Primulaceae), a species endemic to China. *Plant Syst. Evol.* 261 89–98. 10.1007/s00606-006-0430-0

[B39] Julien Y. Sobrino J. A. (2009). Global land surface phenology trends from GIMMS database. *Int. J. Remote Sens.* 30 3495–3513. 10.1080/01431160802562255

[B40] Kao T. C. Cheng C. Y. (1975). Synopsis of the Chinese Rheum. *Acta Phytotax. Sin.* 13 69–82.

[B41] Kaur G. Singh B. P. Nagpal A. K. (2013). Phenology of Some Phanerogams (Trees and Shrubs) of North-western Punjab, India. *J. Bot.* 2013:712405. 10.1155/2013/712405

[B42] Khajuria A. (2013). *Conservation Biology of Three Overexploited Medicinal Plants of North-West Himalayan Region*. Ph.D. thesis. Rajouri: Baba Ghulam Shah Badshah University.

[B43] Khajuria A. Verma S. Sharma P. (2011). Stylar movement in *Valeriana wallichii* DC.- a contrivance for reproductive assurance and species survival. *Curr. Sci.* 100 1143–1144.

[B44] Khan S. Kumari P. Wani I. A. Verma S. (2021). Pollination biology and breeding system of *Vitex negundo* L. (Lamiaceae), an important medicinal plant. *Int. J. Plant Rep. Biol.* 13 77–82.

[B45] Korner C. (2003). *Alpine Plant Life*, 2nd Edn. (Heidelberg: Springer), 101–119.

[B46] Korner C. (2007). The use of ‘altitude’ in ecological research. *Trends Ecol. Evol.* 22 569–574. 10.1016/j.tree.2007.09.006 17988759

[B47] Korner C. Cochrane P. M. (1983). Stomatal responses and water relations of Eucalyptus pauciflora in summer along an elevational gradient. *Oecologia* 66 443–455. 10.1007/BF00378313 28310877

[B48] Korner C. Neumayer M. Menendez-Riedl S. P. Smeets-Scheel A. (1989). Functional morphology of mountain plants. *Flora* 182 353–383.

[B49] Kumari P. Khajuria A. Wani I. A. Khan S. Verma S. (2020). Effect of floral size reduction on pollination and reproductive efficiency of female flowers of Valeriana wallichii, a threatened medicinal plant. *Natl. Acad. Sci. Lett.* 44 75–79. 10.1007/s40009-020-00954-8

[B50] Lewis D. (1979). *Sexual Incompatibility in Pants.* London: Edward Arnold Publishers limited.

[B51] Li A. R. (1998). *Flora Republicate Popularis Sinicae.* Beijing: Science press.

[B52] Li I. Y. Kleunen M. V. Stift M. (2019). Sibling competition does not magnify inbreeding depression in North American *Arabidopsis lyrata*. *Heredity* 123 723–732. 31541202 10.1038/s41437-019-0268-1 PMC6834581

[B53] Li Q. J. Xu Z. F. Kress W. J. Xia Y. M. Zang L. Deng X. B. (2001). Flexible style that encourages outcrossing. *Nature* 410 431–431. 10.1038/35068635 11260703

[B54] Li T. Liu X. Li Z. Ma H. Wan Y. Liu X. (2018). Study on Reproductive Biology of Rhododendron longipedicellatum: a Newly Discovered and Special Threatened Plant Surviving in Limestone Habitat in Southeast Yunnan, China. *Front. Plant Sci.* 9:33. 10.3389/fpls.2018.00033 29445383 PMC5797782

[B55] Linnaeus C. (1753). *Rheum ribes”. Species Plantarum, Tomus I.* Stockholm: Impensis Laurentii Salvii.

[B56] Mayer C. Adler L. Armbruster W. S. Dafni A. Eardley C. Huang S.-Q. (2011). Pollination ecology in the 21st century: key questions for future research. *J. Pollinat. Ecol.* 3 8–23. 10.26786/1920-7603(2011)1

[B57] Mercado J. A. Fernfindez-Mufioz R. Quesada M. A. (1994). In vitro germination of pepper pollen in liquid medium. *Sci. Hortic.* 57 273–281.

[B58] Moore L. M. Lauenroth W. K. (2017). Differential effects of temperature and precipitation on early- vs. late-flowering species. *Ecosphere* 8:e01819. 10.1002/ecs2.1819

[B59] Moza K. M. Bhatnagar A. K. (2007). Plant reproductive biology studies crucial for conservation. *Curr. Sci.* 92:1207.

[B60] Mulik N. G. Bhosale L. J. (1989). Flowering phenology of the mangroves from the West Cost of Maharashtra. *J. Bombay Nat. Hist. Soc.* 3 355–339.

[B61] Nautiyal B. P. Nautiyal M. C. Rawat N. Nautiyal A. R. (2009). Reproductive biology and breeding system of Aconitum balfourii (Benth) Muk: a high altitude endangered medicinal plant of Garhwal Himalaya, India. *Res. J. Med. Plants* 3 61–68. 10.3923/rjmp.2009.61.68

[B62] Neal P. Anderson G. J. (2005). Are mating system breeding system of inconsistent and confusing terminology in plant reproductive biology. Or is the other way around. *Plant Syst. Evol.* 2501 173–185. 10.1007/s00606-004-0229-9

[B63] Nebot A. Cogoni D. Fenu G. Bacchetta G. (2016). Floral biology and breeding system of the narrow endemic *Dianthus morisianus* Vals. (Caryophyllaceae). *Flora* 219 1–7. 10.1016/j.flora.2015.12.004

[B64] Noy-Porat T. Flaishman M. A. Eshel A. Sandler-Ziv D. Kamenetsky R. (2009). Florogenesis of the Mediterranean geophyte *Narcissus tazetta* and temperature requirements for flower initiation and differentiation. *Sci. Hortic.* 120 138–142. 10.1016/j.scienta.2008.09.016

[B65] Ollerton J. (2014). Sunbird surprise for syndromes. *Nature* 394 726–727. 10.1038/29409

[B66] Pando J. B. Tchuenguem F. N. Tamesse J. L. (2011). Foraging and pollination behaviour of *Xylocopacalens lepeletier* (Hymenoptera: Apidae) on *Phaseolus coccineus* L. (Fabaceae) flowers at Yaounde (Cameroon). *Entomol. Res.* 41 185–193. 10.1111/j.1748-5967.2011.00334.x

[B67] Phillips N. C. Drost D. T. Varga W. A. Shultz L. M. (2011). Demography, reproduction, and dormancy along altitudinal gradients in three intermountain *Allium* species with contrasting abundance and distribution. *Flora* 206 164–171. 10.1016/j.flora.2010.05.002

[B68] Pluess A. R. Frei E. Kettle C. J. Hahn T. Ghazoul J. (2011). Plant growth and fitness of *Scabiosa columbaria* under climate warming conditions. *Plant Ecol. Divers.* 4 379–389. 10.1080/17550874.2011.618848

[B69] Porch T. G. Jahn M. (2001). Effects of high-temperature stress on microsporogenesis in heat-sensitive and heat-tolerant genotypes of *Phaseolus vulgaris*. *Plant Cell Environ.* 24 723–731.

[B70] Pradervand J. N. Pellissier L. Randin C. F. Gusain A. (2014). Functional homogenization of bumblebee communities in alpine landscapes under projected climate change. *Clim. Change Resp.* 1 1–10.

[B71] Proctor M. Yeo P. Lack A. (1996). *The Natural History of Pollination.* Portland, OR: Timber Press.

[B72] Ramirez N. Nassar J. M. (2017). Breeding systems in Angiosperms: novel inferences from a new analytical approach. *Plant Syst. Evol.* 303 119–137. 10.1007/s00606-016-1357-8

[B73] Rashid S. Kaloo Z. A. Singh S. Bashir I. (2014). Medicinal importance of genus Rheum- A review. *Int. J. Adv. Res.* 2 261–267.

[B74] Rathcke B. Lacey E. P. (1985). Phenological patterns of terrestrial plants. *Annu. Rev. Ecol. Syst.* 6 16–17. 10.1146/annurev.es.16.110185.001143

[B75] R Core Team (2020). *R: A Language and Environment for Statistical Computing.* Vienna: R Foundation for Statistical Computing.

[B76] Rodriguez-Riano T. Dafni A. (2007). Pollen-stigma interference in two gynodioecious species of Lamiaceae with intermediate individuals. *Ann. Bot.* 100 423–431. 10.1093/aob/mcl168 16877457 PMC2735308

[B77] Rohde A. Bhalerao R. (2007). Plant dormancy in the perennial context. *Trends Plant Sci.* 12 217–223. 10.1016/j.tplants.2007.03.012 17416545

[B78] Ruan C. Li H. Mopper S. (2009). Kosteletzkya virginica displays mixed mating in response to the pollinator environment despite strong inbreeding depression. *Plant Ecol.* 203 183–193. 10.1007/s11258-008-9525-8

[B79] Sagoo M. I. S. Farooq U. (2011). Cytology of Rheum, a vulnerable medicinal plant from Kashmir Himalaya. *Chrom. Bot.* 6 41–44. 10.3199/iscb.6.41

[B80] Sajjad A. Saeed S. Muhammad W. Arif M. J. (2009). Role of insects in cross-pollination and yield attributing components of Sesbania sesban. *Int. J. AgriC. Biol.* 11 77–80.

[B81] Schemske D. W. Lande R. (1985). The evolution of self fertilization and inbreeding depression in plants. II. Empirical observations. *Evolution* 39 41–52. 10.1111/j.1558-5646.1985.tb04078.x 28563649

[B82] Shivanna K. R. (2003). *Pollen Biology and Technology.* Enfield: Science Publishers.

[B83] Shivanna K. R. Rangaswamy N. S. (1992). *Pollen Biology - A Laboratory Manual.* Berlin: Springer Verlag.

[B84] Shivanna K. R. Sastri D. C. (1981). Stigma-surface esterase activity and stigma receptivity in some taxa characterized by wet stigmas. *Ann. Bot.* 47 53–64. 10.1093/oxfordjournals.aob.a086000

[B85] Siddique M. A. A. Dar N. A. Wafai B. A. Beigh Y. S. (1997). Reproductive biology of *Podophyllum hexandrum* Royle (Podophyllaceae) an important, rare and threatened Himalayan medicinal plant. *Proc. Natl. Acad. Sci. India Bhubaneshwar* 10–11. 10.15258/sst.2009.37.1.02

[B86] Singh V. K. Barman C. Tandon R. (2014). Nectar Robbing Positively Influences the Reproductive Success of *Tecomella undulata* (Bignoniaceae). *PLoS One* 9:e102607. 10.1371/journal.pone.0102607 25036554 PMC4103821

[B87] Smitha G. R. Thondaiman V. (2016). Reproductive biology and breeding system of *Saraca asoca* (Roxb.) De Wilde: a vulnerable medicinal plant. *Springer Plus* 5 2–15. 10.1186/s40064-016-3709-9 27995002 PMC5125291

[B88] Sreekala A. K. (2017). Importance of plant reproductive biology in conservation. *Paper Presented at the National Conference on “Bioresources: Conservation, Utilization and Future Prospects”GRI-DU*, Gandhigram, 4–5.

[B89] Srinivas G. Babykutty S. Sathiadevan P. P. Srinivas P. (2007). Molecular mechanism of emodin action: transition from laxative ingredient to an antitumor agent. *Med. Res. Rev.* 27 591–608. 10.1002/med.20095 17019678

[B90] Stewart R. R. (1972). *An Annotated Catalogue of Vascular Plants of West Pakistan and Kashmir.* (Karachi: Fakhri Printing Press), 1028.

[B91] Stinson K. A. (2004). Natural selection favors rapid reproductive phenology in *Potentilla pulcherrima* (Rosaceae) at opposite ends of a subalpine snowmelt gradient. *Am. J. Bot.* 91 531–539. 10.3732/ajb.91.4.531 21653408

[B92] Stocklin J. Kuss P. Pluess A. R. (2009). Genetic diversity, phenotypic variation and local adaptation in the alpine landscape: case studies with alpine plant species. *Bot. Helv.* 119 125–133. 10.1007/s00035-009-0065-1

[B93] Tabin S. Kamili A. N. Ganie S. A. Zargar O. Sharma V. Gupta R. C. (2016). Genetic diversity and population structure of Rheum species in Kashmir Himalaya based on ISSR markers. *Flora* 223 121–128. 10.1016/j.flora.2016.05.001

[B94] Tali B. A. Ganie A. H. Nawchoo I. A. Wani A. A. Reshi Z. A. (2014). Assessment of threat status of selected endemic medicinal plants using IUCN regional guidelines: a case study from Kashmir Himalaya. *J. Nat. Conserv.* 23 80–89. 10.1016/j.jnc.2014.06.004

[B95] Tandon R. Shivanna K. R. Mohan-Ram H. Y. (2003). Reproductive biology of *Butea monosperma* (Fabaceae). *Ann. Bot.* 92 715–723. 10.1093/aob/mcg193 14500327 PMC4244857

[B96] Tao L. X. Tan H. J. Wang X. Cao L. Y. Song J. Cheng S. H. (2008). Effects of high temperature stress on lowering and grain-setting characteristics for Guodao 6. *Acta Agron. Sin.* 34 669–674. 10.3724/sp.j.1006.2008.00669

[B97] Ved D. K. Kinhal G. A. Ravi Kumar K. Prabhakaran V. Ghate U. Vijaya Shankar R. (2003). *Conservation Assessment and Management Prioritization for the Medicinal Plants of Jammu & Kashmir, Himachal Pradesh & Uttaranchal.* Bangalore: Foundation for Revitalisation of Local Health Traditions.

[B98] Verma S. Kaul V. Magotra R. Koul A. K. (2008). Pollinator induced anther dehiscence in *Incarvillea emodii* (Bignoniaceae). *Curr. Sci.* 94 1372–1374.

[B99] Verma S. Magotra R. Koul A. K. (2003). Restoration of *Eremostachys superba* Royle ex Benth.- a critically endangered species. *Curr. Sci.* 84 1307–1308.

[B100] Verma S. Magotra R. Koul A. K. (2004). Stylar movement avoids self and promotes cross-pollination in *Eremurus himalaicus*. *Curr. Sci.* 87 872–873.

[B101] Vikas T. R. (2011). Reproductive biology of *Azadirachta indica* (Meliaceae), a medicinal tree species from arid zones. *Plant Species Biol.* 26 116–123. 10.1111/j.1442-1984.2010.00311.x

[B102] Wani I. A. Verma S. Kumari P. Charles B. Hashim M. J. El-Serehy H. A. (2021). Ecological assessment and environmental niche modelling of *Himalayan rhubarb* (*Rheum webbianum* Royle) in northwest Himalaya. *PLoS One* 16:e0259345. 10.1371/journal.pone.0259345 34793481 PMC8601538

[B103] Yamasaki E. Sakai S. (2013). Wind and insect pollination (ambophily) of *Mallotus* spp. (Euphorbiaceae) in tropical and temperate forests. *Aust. J. Bot.* 61 60–66. 10.1071/bt12202

[B104] Yaqoob U. Nawchoo I. A. (2015). Impact of habitat variability and altitude on growth dynamics and reproductive allocation in Ferula jaeschkeana Vatke. *J. King Saud Univ. Sci.* 29 1–9. 10.1016/j.jksus.2015.10.002

[B105] Yaqoob U. Nawchoo I. A. (2016). Reproductive ecology of an endangered monocarpic herbaceous perennial, *Ferula jaeschkeana* Vatke. *Trop. Ecol.* 57 849–864.

